# A Framework to Establish Diet and Nutrition Competencies for Oral Health Care Education

**DOI:** 10.1002/jdd.70127

**Published:** 2025-12-17

**Authors:** Teresa A. Marshall, Michael Crowe, Riva Touger‐Decker

**Affiliations:** ^1^ Department of Preventive and Community Dentistry College of Dentistry University of Iowa Iowa City Iowa USA; ^2^ Dublin Dental University Hospital Dublin Ireland; ^3^ Rutgers School of Health Professions Rutgers, The State University of New Jersey Newark New Jersey USA

**Keywords:** competency, diet, nutrition, oral health

## Abstract

**Objective:**

To prevent and manage oral disease, oral health care practitioners (OHCPs) must provide dietary counseling based on nutrition science. OHCPs are often ill‐equipped to provide such counseling due to fragmented and inadequate dietary education, which is typically attributed to limited curricular time or appropriately qualified faculty. Perhaps a more significant barrier is the absence of agreement on diet and nutrition competencies to guide oral health curricular content. Our objective was to define a framework of diet and nutrition competencies for the graduating oral health care student.

**Methods:**

Initially, we identified the core diet and nutrition knowledge and associated educational objectives necessary to facilitate effective dietary counseling to support oral health. Subsequently, we identified behavioral expectations with evaluation criteria for diet and nutrition oral health competencies.

**Results:**

Diet and nutrition oral health domains secondary to food choices, dietary behaviors, and/or nutrient intakes included caries, erosion, periodontal disease, and oral cancer, while outcomes of oral disease impacting food choices and dietary behaviors included oral dysfunction. Fundamental diet and nutrition principles supporting oral and systemic health and counseling skillsets were identified. Behavioral expectations for OHCPs for each oral health domain were articulated. For example, the behavioral expectation for caries competency is “Identifies and addresses diet‐related caries risk factors within the context of environmental and social barriers.”

**Conclusions:**

A framework to identify the knowledge base for and define diet and nutrition oral health competencies is presented as a foundation to advance diet and nutrition‐related oral health education.

## 1 Introduction

According to the World Health Organization (WHO), oral health is defined as having a mouth, teeth, and orofacial structures that enable oral function (i.e., smiling, eating, and speaking), and support psychosocial dimensions (i.e., self‐confidence, well‐being, and freedom from pain or embarrassment) [1]. The WHO recognizes that oral diseases are largely preventable and share risk factors with other non‐communicable diseases. Both oral and systemic diseases are associated with social determinants of health and attributed to health inequities throughout the world [2]. Furthermore, oral and systemic diseases share common dietary risk factors, with a well‐balanced diet low in added sugars playing a preventive role in both [1, 3, 4, 5].

Although the terms diet, nutrient, and nutrition are often used interchangeably, they have different definitions. Diet refers to foods and beverages consumed; nutrients to the compounds within foods and beverages that provide energy and regulate growth, maintenance, and repair of body tissues; and nutrition to the science of nutrients within foods and beverages and their assimilation within the body.

Relationships between healthy diets and both oral and systemic disease have been studied extensively during the past 75 years. Oral health care practitioners (OHCPs) have long recognized the roles of fermentable carbohydrates in the caries process and dietary acids in dental erosion. Associations between food choices, nutrient intakes, and alcohol exposure and periodontal disease and oral cancers have been identified more recently. The impact of oral dysfunction with or without orofacial pain on food and nutrient intakes and subsequent risk of malnutrition across the lifespan, but especially during aging, has received less attention. It is increasingly being acknowledged that oral and systemic disease share common dietary risk factors. Furthermore, systemic diseases related to lifestyle habits, including diet (i.e., obesity, Type 2 diabetes, metabolic syndrome, and cardiovascular disease), are thought to increase the risk of and/or exacerbate periodontal disease.

Diet, nutrition, and oral health are integrally related through multidirectional associations. If OHCPs are serious about preventing and managing oral diseases, then they need to acknowledge these relationships and address their patients’ diets to facilitate oral and systemic health. To do so, the OHCPs must understand diet and nutrient‐related oral health domains, basic diet and nutrition principles, and dietary counseling strategies. Unfortunately, diet and nutrition education within dental and dental hygiene schools is often fragmented and incomplete [6, 7]. The limited diet and nutrition education of oral health care students (OHCS) is nothing new; Touger‐Decker reported that nutrition hours for dental students ranged between 14.7 and 16 h between 1985 and 1997 [8]. Interestingly, she reported that clinical (counseling and assessment) were greater than classroom (knowledge) hours [8]. In 2013, the American Dietetic Association's position paper on oral health and nutrition suggested that interprofessional collaboration between dietetics and dental professionals is necessary to support oral health [9].

More recently, a scoping review conducted by Fernández et al. reported that while the importance of diet and nutrition in oral health is acknowledged, barriers to diet and nutrition education, including curricular time, limited qualified faculty including faculty dietitians, and insufficient interprofessional collaboration, prevent educational advancement [6]. Fernández et al. also identified the lack of a standardized curriculum as a barrier [6]. Fundamental to a standardized curriculum is competency criteria—that is, what do we want the graduating OHCSs to be able to do? Identification of diet and nutrition‐related oral health competency criteria and development of educational strategies are needed. Finally, acknowledgement of the importance of diet and nutrition in oral health by accrediting bodies (i.e., CODA) is essential to encourage inclusion in dental schools.

The overall objective of this project was to define a framework of diet and nutrition‐related oral health competencies for the graduating OHCS to promote oral, systemic, and nutritional health consistent with WHO's vision of oral health. To achieve this objective, we first identified the core diet and nutrition knowledge necessary to facilitate effective diet assessment and counseling to support oral, systemic, and nutritional health.

## 2 Methods

### 2.1 Research Team

The research team was composed of three faculty members from two different dental schools in the United States and one dental school in Ireland. All three faculty have doctoral degrees in human nutrition; two are registered dietitians and one is a dentist. Each team member has been engaged in diet‐related dental education and research throughout their careers and has presented their individual diet‐related oral health curricula at international meetings (i.e., International Association for Dental Research in New Orleans 2024 and Association for Dental Education in Europe in Leuven, 2024).

### 2.2 Process

To establish a competency framework, the research team addressed three foundational questions to identify the core diet and nutrition knowledge necessary to facilitate effective dietary counseling for oral, systemic, and nutritional health.

1. What are the diet‐ and nutrient‐related oral health domains? To address this question, we identified:
  - Oral diseases secondary to food choices, dietary behaviors, and nutrient intakes.
  - Outcomes of oral disease impacting food choices, dietary behaviors, and nutrient intakes.
2. What are the diet and nutrition principles supporting diet‐ and nutrient‐related oral health domains and systemic health?
3. What are the behavioral concepts necessary to support dietary counseling for oral health? Within this question, we identified:
  - Screening and assessment protocols appropriate to identify diet‐ and nutrient‐related oral risk factors.
  - Counseling skillsets appropriate to facilitate diet‐related behaviors to reduce oral disease.

This sequential approach ensured that competencies were grounded in disease relationships (Q1), supported by scientific principles (Q2), and operationalized through behavioral skills (Q3).

Following identification of the core diet and nutrition knowledge, we articulated the diet‐ and nutrition‐related oral health competencies for the graduating OHCS. We initially identified educational objectives according to Bloom's knowledge and cognitive process domains [10]. Following identification of the educational objectives, we identified the behavioral expectations and evaluation criteria to establish diet‐related oral health competencies [11].

## 3 Results

### 3.1 Diet‐ and Nutrient‐Related Oral Health Domains

Knowledge of the science supporting associations between dietary behaviors and oral disease, as well as knowledge of how oral dysfunction impacts food choices, is necessary for the OHCPs to rationalize dietary counseling.

Four oral diseases associated with food choices, dietary behaviors, and/or nutrient intakes were identified. First, the dental caries process is considered a diet‐dependent disease as the presence of fermentable carbohydrates, primarily as added sugars, is necessary for caries development; dietary behaviors impacting exposure time moderate the process [12, 13]. Second, dental erosion may be associated with prolonged exposure to acidic foods and/or beverages [14, 15]. Third, periodontal disease is a nutrient‐related disease as tissue health, a functional immune system, and the tissue's response to periodontal therapy require adequate energy and nutrient intakes; lower overall diet quality and individual nutrient deficiencies have been associated with increased risk of periodontal disease [16, 17, 18]. Finally, oral cancer is associated with high intakes of carcinogenic food compounds and alcohol and low intakes of fruits, vegetables, and micronutrients [19, 20, 21]. Understanding how food and beverage choices impact oral disease risk facilitates the provision of dietary recommendations to reduce oral disease risk.

Oral outcomes, including edentulism, denture use, orofacial pain, xerostomia, hyposalivation, and loss of soft tissue secondary to oral disease, contribute to oral dysfunction [22]. In turn, oral dysfunction with or without dysphagia leads to modification of food choices with implications for energy and nutrient intakes and subsequently risk of malnutrition [23, 24]. Recognizing how oral outcomes might impact food choices and dietary behaviors is a prerequisite to providing anticipatory guidance to facilitate texture and consistency modification to ensure appropriate energy and nutrient intakes.

### 3.2 Diet and Nutrition Principles Supporting Diet‐ and/or Nutrient‐Related Oral Health Domains and Systemic Health

Knowledge of core diet and nutrition principles, diet therapy for systemic disease, and public health nutrition is necessary for the OHCPs to assess the diet and provide dietary counseling consistent with diet‐related systemic disease prevention and nutritional health to reduce oral disease.

#### 3.2.1 Dietary Guidance

Dietary guidelines, including the United States Department of Agriculture's Dietary Guidelines for Americans (USDGA) provide dietary guidance for healthy individuals [25]. The USDGA is the foundation for food‐related policy (i.e., school lunch programming, Supplemental Nutrition Assistance Program) [25], recommends food intakes to promote adequate nutrient intakes and limit diet‐related chronic disease (i.e., MyPlate) [26], and identifies nutrient requirements (i.e., Dietary Recommended Intakes) [27].

Food groups (i.e., fruits, vegetables, grains, proteins, and dairy) have different macronutrient (i.e., carbohydrate, fat, and protein) and micronutrient (i.e., vitamin, mineral) compositions, which inform the recommended intakes for each food group to ensure adequate nutrient intakes within energy requirements. Deviation from recommended food group intakes, as well as ultra‐processed food choices, are associated with oral and systemic diet‐related diseases. Knowledge of carbohydrate composition (i.e., starch, processed starches, and sugars, particularly added or free sugars) within food groups facilitates food guidance for caries prevention. In addition, understanding food science principles related to low‐calorie and non‐nutritive sweetener use, functionality, and cariogenicity within food and beverage products facilitates recommendations for caries prevention.

Beyond what is consumed, how foods and beverages are consumed is associated with the risk of diet‐related oral and systemic disease. Knowledge of dietary behaviors supporting defined meal patterns, appropriate meal and snack structure, and limited adverse exposures is necessary to optimize dietary recommendations.

Popular or fad (i.e., keto, gluten‐free, Atkins, paleo) diets targeting weight loss and/or with perceived health benefits are often promoted by celebrities, influencers, and community leaders. Because such diets restrict food choices, they are often energy‐ and nutrient‐deficient, which can increase the risk of oral and systemic diseases. Some popular diets promote acidic foods (i.e., the grapefruit diet), which can increase the risk of erosion and perhaps caries interactions. Knowledge of common popular diets is necessary to facilitate dietary guidance for oral and systemic health.

#### 3.2.2 Diet‐Related Systemic Disease

The risk of multiple chronic systemic diseases, including obesity, cardiometabolic diseases (i.e., cardiovascular disease, hypertension, Type 2 diabetes, and metabolic syndrome), and cancers, is associated with excess energy intake, altered macronutrient intakes, and micronutrient deficiencies. The USDGA is designed to reduce the risk of diet‐related systemic disease [25]. Thus, compliance with MyPlate, consistent with energy requirements, is recommended to prevent systemic disease [26]. Knowledge of MyPlate dietary recommendations enables the OHCPs to provide dietary recommendations for oral health consistent with systemic and nutritional health.

While noncompliance with dietary recommendations similarly contributes to both oral and systemic disease, systemic disease impacts oral disease through additional mechanisms. For example, the accumulation of excess adipose tissue is inflammatory; chronic inflammation secondary to obesity is associated with an increased risk of periodontal disease [28]. As another example, individuals with vomiting behaviors associated with either gastroesophageal reflux or eating disorders are at risk of erosion. Knowledge of systemic—oral disease relationships is necessary for the OHCPs to consider systemic disease related risk factors for oral disease and provide anticipatory dietary guidance to lower risk of oral disease.

Medical nutrition therapy (MNT) is defined as individualized dietary programming designed to treat systemic conditions adversely impacted by normal nutrition recommendations. For example, several inborn errors of metabolism, including phenylketonuria require strict limitation of specific nutrients (i.e., phenylalanine) due to the metabolic error [29]. In addition, oral manifestations of systemic disease might indicate a need for MNT. For example, oral complications of celiac disease include recurrent aphthous ulcers, enamel defects, and delayed tooth eruption in children. Celiac disease is an autoimmune disorder in which gluten (a protein found in wheat) damages the small intestine leading to nutrient malabsorption and the associated oral complications [30]. MNT for celiac disease requires gluten avoidance and micronutrient status monitoring. While MNT is generally provided by registered dietitians and beyond the OHCP's scope of practice, awareness of common conditions and their respective dietary therapies and oral health implications is necessary to ensure that diet‐related oral health recommendations are consistent with MNT.

#### 3.2.3 Public Health Nutrition

Food choices and dietary behaviors are driven by complex interactions between individual, social, cultural, and economic factors within one's lived environment. The discipline of public health nutrition addresses diet‐related inequities to prevent diet‐related disease and promote nutritional health across all sectors of the community. As part of a community, OHCPs must be aware of food and nutrition policies and barriers to food and nutrition security to facilitate a healthy food environment in which their patients can make healthy food choices.

Food security is defined as having access to adequate and appropriate foods and beverages to live a healthy lifestyle, while nutrition security expands this definition to ensure access to foods and beverages that promote well‐being and prevent diet‐related chronic disease. Food sufficiency addresses hunger within health disparities and nutrition security leads to health equity [31]. Multiple governmental agencies are charged with promoting food safety and preventing foodborne illness to ensure that available foods do not cause harm. Knowledge of food and nutrition security and food safety is necessary for the OHCPs to provide reasonable dietary recommendations and address barriers to healthy foodstuff within their communities.

Sustainable healthy diets (SHDs), as defined by the Food and Agriculture Organization and World Health Organization, are dietary patterns that promote health and well‐being while having a low environmental impact [32]. Understanding SHD principles is increasingly relevant for OHCPs, as many evidence‐based oral health recommendations align with environmental sustainability goals. For example, reducing added sugar intake for caries prevention also supports sustainability, as sugar production is associated with substantial greenhouse gas emissions and intensive water consumption [33, 34]. Knowledge of these intersections enables OHCPs to provide dietary counseling that addresses both individual oral health and broader public health considerations.

Nutrient fortification is defined as the addition of nutrients not originally present to the consumed product, and enrichment is defined as the addition of nutrients removed during processing to the consumed product. With respect to oral health, vitamin D fortified cow's milk is a significant source of dietary vitamin D; vitamin D deficiencies during tooth development are associated with early childhood caries [35]. Furthermore, folic acid fortified grains may play a role in reducing cleft lip and palate in infants [36]. Thus, awareness of public health efforts to reduce nutrient deficiencies is necessary for the OHCPs to advocate for oral health promotion.

Food and nutrient misinformation and disinformation are rampant in contemporary society, being readily distributed by influencers through online platforms. While misinformation might be less damaging than disinformation, both can contribute to physical, psychosocial, and financial harm. OHCPs faced with diet‐related misinformation and disinformation require a scientifically accurate diet and nutrition foundation, as well as strategies and resources to facilitate patient education consistent with nutritional health to reduce the risk of oral and systemic disease.

### 3.3 Behavioral Concepts Necessary to Support Dietary Counseling for Oral Health

Knowledge of dietary assessment, counseling, and resource referral principles and clinical experience assessing diets and counseling patients are necessary to prepare the OHCPs to provide such services in clinical practice.

Dietary screening protocols query key dietary risk factors for oral and/or systemic disease and barriers to achieving healthy diets. Risk factors might include the quantity, frequency, and duration of sugar‐sweetened beverage intakes, meal structure, compliance with MyPlate age and gender specific recommendations, and food security status [13]. Dietary assessment of individuals screening positive for risk factors or presenting with obvious diet‐related oral disease requires more detailed analyses of food and beverage choices, eating behaviors, and resource availability. Following identification of diet‐related risk factors, dietary counseling to understand the patient's perspective of their risk factors, as well as their desire and capacity to modify the risk factors, provides a framework to negotiate dietary recommendations to improve oral, systemic, and nutritional health.

For patients to act on dietary recommendations, the recommendations must be actionable. In other words, the patient must have the knowledge and culinary skillset to select and prepare appropriate foods within their budget from food outlets within their environment. Addressing patient knowledge and skillset limitations is beyond the scope of practice of the OHCPs. Thus, awareness of government and private food programs, social service organizations, and registered dietitians within the community is necessary for the OHCPs to refer patients for help to comply with oral health‐related dietary recommendations. Collaboration with registered dietitians to develop diet‐related oral health recommendations may be necessary for patients with MNT.

Clinical experiences that is, opportunities for OHCS to screen and assess diets using appropriate software and provide dietary counseling, are necessary to facilitate comfort in doing so. Early experiences evaluating their own or peers’ diets are appropriate prior to evaluating patient diets on the clinic floor.

### 3.4 Diet‐ and Nutrition‐Related Oral Health Competencies

Following identification of the core diet and nutrition knowledge, educational objectives were organized according to Bloom's knowledge and cognitive process dimensions (Table 1). The educational objectives for diet‐related oral health domains are identified in Table 1a. Considering dental caries, the educational objective ‘identify fermentable carbohydrates within diet’ depends on the cognitive process of remembering within the factual knowledge dimension “definition and nature of fermentable carbohydrates.” The educational objectives are defined at the conceptual as opposed to the granular level, as observed with course or lecture learning outcomes. Educational objectives for diet and nutrition principles and dietary counseling are identified in Table 1b.

**TABLE 1:** Educational objectives for a dietary oral health curriculum organized according to Bloom's knowledge and cognitive process dimensions.

	Bloom's knowledge dimension	Bloom's cognitive process dimension	Educational objectives
**(a) Diet‐related oral health domains**			
Dental caries	*Factual*: Mechanism of caries process; Definition and nature of fermentable carbohydrates; Definition of exposure time	*Remember*: Recognizing and recalling *Understand*: Interpreting and inferring	a. Identify fermentable carbohydrates within diet b. Quantify length of exposure to fermentable carbohydrates
	*Conceptual*: Relationships among food choices, eating behaviors, oral function, and social determinants of health	*Understand*: Interpreting, summarizing, and inferring *Analyze*: Differentiating	a. Identify caries risk based on concurrent food composition (i.e., protein, fat, and fiber) b. Distinguish between frequency and length of carbohydrate exposure c. Distinguish between structured and unstructured meal patterns d. Describe how oral dysfunction influences food choices due to both function and sensory impacts e. Identify barriers (i.e., knowledge, financial, access) to healthy food choices
	*Procedural*: Diet/nutrition assessment within scope of practice; Dietary counseling within scope of practice	*Apply*: Executing	a. Screen healthy patients for diet‐related caries risk b. Assess diet of healthy patients who fail diet‐related caries risk screen and patients with active caries c. Counsel patients to reduce diet‐related caries risk and support systemic health d. Refer patients for dietary counseling beyond scope of practice and/or with resource barriers
	*Metacognitive*: Integration of patient food choices, eating behaviors, oral function, and social determinants of health within environmental context with oral findings	*Create*: Assembling and planning	a. Identify the relevance of patient food choices and eating behaviors in caries risk profile b. Identify resources and/or strategies (including referrals) to address barriers to healthy eating behaviors c. Outline a dietary plan to reduce caries risk
Dental erosion	*Factual*: Mechanism of erosion process; Dietary sources of acid exposure; Systemic conditions associated with oral acid exposure	*Reme* *mber*: Recognizing and retrieving *Understand*: Interpreting and classifying	a. Identify sources of dietary acid b. Identify systemic conditions associated with oral acid exposure
	*Conceptual*: Eating behaviors prolonging acidic food exposure; Relationships among disordered eating (including anorexia nervosa and bulimia nervosa), food choices, and oral outcomes	*Remember*: Recognizing *Analyze*: Differentiating and attributing	a. Identify eating behaviors prolonging dietary acid exposures contributing to erosion b. Identify behaviors associated with disordered eating contributing to erosion
	*Procedural*: Dietary counseling within scope of practice; Refer for suspected eating disorders	*Apply*: Executing	a. Screen for presence of an eating disorder b. Refer patients with suspected eating disorder to appropriate health care professional c. Counsel patients to reduce erosion associated with food and/or beverage choices and/or eating behaviors
	*Metacognitive*: Integration of medical history, dietary behaviors, and oral findings	*Create*: Generating, planning	a. Outline alternative eating behaviors to reduce erosion risk
Periodontal disease	*Factual*: Mechanism of nutrients in tissue health and immune function; Associations between diet‐related systemic chronic disease and periodontal disease	*Remember*: Recognizing and retrieving *Understand*: Interpreting and inferring	a. Identify significance of adequate nutrient intakes for tissue health and immune function b. Identify associations between diet‐related chronic disease and periodontal disease
	*Conceptual*: Relationships among food choices, nutrient intakes and periodontal disease; Relationships between diet‐related systemic conditions and periodontal disease; Relationships among food choices, oral function, and social determinants of health	*Understand*: Interpreting and inferring *Analyze*: Differentiating and attributing	a. Recognize associations between overall diet quality and/or individual nutrient intakes and periodontal disease b. Recognize influence of diet‐related systemic chronic disease on periodontal disease c. Describe how oral dysfunction influences food choices d. Identify barriers (i.e., knowledge, financial, access) to healthy food choices
	*Procedural*: Diet/nutrition assessment within scope of practice; Dietary counseling within scope of practice	*Apply*: Executing	a. Screen and/or assess patient diets for food compliance with dietary guidelines b. Counsel patients to reduce diet‐related periodontal disease risk and support systemic health c. Refer patients for dietary counseling beyond scope of practice and/or with resource barriers
	Metacognitive: Integration of patient food choices, oral function, and social determinants of health within the environmental context with oral findings	*Create*: Assembling and planning	a. Identify the relevance of patient food choices for periodontal disease risk b. Identify the relevance of patient food choices for systemic chronic disease and their impact on periodontal disease risk c. Identify resources and/or strategies (including referrals) to address barriers to healthy eating behaviors d. Outline a dietary plan to reduce periodontal disease risk and support systemic health
Oral cancers	Factual: Associations between inadequate food (i.e., citrus and dark green vegetables) and/or nutrient intakes, and oral cancers; Associations between excess alcohol, fried foods, and oral cancer	*Remember*: Remembering and retrieving *Understand*: Interpreting and inferring	a. Identify significance of adequate nutrient intakes for tissue health and cancer prevention b. Identify associations between alcohol, carcinogenic compounds within the diet, and oral cancer
	*Conceptual*: Relationships among food choices, nutrient intakes, and oral cancer; Relationships among food choices, oral function, and social determinants of health	*Understand*: Interpreting and inferring *Analyze*: Differentiate	a. Recognize associations between overall diet quality and individual nutrient intakes and oral cancer b. Describe how oral dysfunction influences food choices c. Identify barriers (i.e., knowledge, financial, access) to healthy food choices
	*Procedural*: Diet/nutrition assessment within scope of practice; Dietary counseling within scope of practice	*Apply*: Executing	a. Screen patients for food compliance with dietary guidelines b. Counsel patients to reduce diet‐related oral cancer risk and support systemic health c. Refer patients for dietary counseling beyond scope of practice and/or with resource barriers
	*Metacognitive*: Integration of patient food choices, oral function, and social determinants of health within environmental context with oral findings	*Create*: Assembling and planning	a. Identify the relevance of patient food choices for oral cancer risk b. Identify resources and/or strategies (including referrals) to address barriers to healthy eating behaviors c. Outline a dietary plan to reduce oral cancer risk
Oral dysfunction	Factual: Mechanism of eating process (e.g., biting, chewing, bolus formation, and swallowing); Impact of oral dysfunction (e.g., tooth loss, denture use, soft tissue loss/disease/pain, pain, xerostomia, hyposalivation, and/or dysphagia) on eating process	*Remember*: Recognizing and recalling *Understand*: Interpreting and inferring	a. Identify oral steps involved in eating solid foods and drinking liquids b. Describe how oral dysfunction impacts food choice c. Differentiate between functional and sensory impacts
	*Conceptual*: Relationship between oral dysfunction, food choice, and nutrient intakes; Adaptive eating strategies for oral dysfunction	*Understand*: Interpreting, summarizing, and inferring *Analyze*: Differentiate	a. Describe how changes in food/beverage intake secondary to oral dysfunction impact energy and nutrient intakes b. Outline how oral dysfunction increases the risk of malnutrition c. List adaptive eating strategies matching the nature of oral dysfunction to improve healthy food/beverage intakes. Love this
	*Procedural*: Evaluation of eating skillset; Dysphagia screening; Food texture and beverage thickness recommendations within scope of practice	*Apply*: Executing *Analyze*: Differentiating and attributing	a. Identify oral dysfunction impacting eating skillset and/or food intake b. Offer adaptive eating guidance including texture modification to facilitate improved dietary intake c. Screen for dysphagia d. Refer for dysphagia assessment and/or adaptive eating therapies
	*Metacognitive*: Integration of oral dysfunction's impact on eating process; Identification of strategies to minimize impact of oral dysfunction on food intake	*Understand*: Classifying and comparing *Evaluate*: Detecting *Create*: Assembling and planning	a. Propose adaptive eating strategies to facilitate food/beverage intakes consistent with dietary guidelines and to support systemic health
**(b) Diet and nutrition principles and dietary counseling**			
Dietary guidelines	*Factual*: Normal nutrition (e.g., food guides, nutrient requirements); Food composition (e.g., nature of carbohydrate) Dietary behaviors Food texture modifications Popular diets	*Remember*: Recognizing and retrieving *Understand*: Interpreting and classifying	a. Articulate recommendations for health promotion, food group intakes, and nutrient requirements b. Identify macronutrient content of food groups with emphasis on added/free sugars, modified starches, and starches c. Describe the effect of food processing on food composition (including use of nonnutritive sweeteners) d. Define meal structure and recommended meal/snack patterns e. Identifies food texture modifications to facilitate eating f. Describe popular diets and their consistency with dietary guidelines
	*Conceptual*: Classification of dietary patterns, food groups, and nutrients; Relationships among food processing, food composition, and oral/systemic disease risk; Relationships among food textures, beverage thickness, and eating safety and ease Relationships among popular diets, nutrient intakes, and oral/systemic disease risk	*Remember*: Recognizing *Understand*: Classifying, interpreting, and explaining *Analyze*: Differentiating and attributing	a. Distinguish between diets, foods, and nutrients b. Describe how food processing modifies food composition with implications for both oral and systemic disease risk c. Describe how modification of food textures and beverage consistencies can facilitate food/beverage intake with oral dysfunction d. Identify consistency between popular diets and dietary guidelines
	*Procedural*:		
	*Metacognitive*: Hypothesize impact of food processing on nutrient intake and both oral/systemic disease risk	*Create*: Generating, planning	a. Predict oral/systemic disease risk based on impact of food processing on food composition b. Predict oral disease risk associated with popular diets
Diet‐related systemic disease	*Factual*: Dietary risk factors for systemic disease; Dietary recommendations for systemic disease prevention and treatment; Diet‐related systemic disease as a risk factor for oral disease; Metabolic disorders and autoimmune disorders requiring diet therapy	*Remember*: Recognizing and retrieving *Understand*: Interpreting and inferring	a. Describe dietary risk factors for systemic disease b. Identify dietary recommendations for systemic disease c. Identify obesity and cardiometabolic disease as risk factors for periodontal disease d. Recognize diet therapy as component of management for some medical conditions
	*Conceptual*: Relationships among food choices, nutrient intakes, and systemic diseases; Consistency of diet‐related risk factors for both oral and systemic disease	*Understand*: Interpreting and inferring *Analyze*: Differentiating and attributing	a. Recognize associations between overall diet quality and/or individual nutrient intakes and systemic disease b. Identify diet‐related risk factors common to both oral and systemic disease
	*Procedural*:		
	*Metacognitive*: Synthesis of inadequate and/or excessive food and/or nutrient intakes with risk of oral/systemic disease	*Create*: Assembling and planning	a. Identify the relevance of patient food choices for both oral and systemic disease risk
Public health nutrition	*Factual*: Food resource programs; Associations between food security, diet quality, and oral disease; Associations between food policies (e.g., vitamin D and folic acid fortification) and malnutrition‐related disorders; Sugar's policies (e.g., labeling, advertising) Nutrition misinformation/health fraud	*Remember*: Recognizing and retrieving *Understand*: Interpreting and inferring	a. Identify referral sources for food access b. Describe associations between food security status and oral disease c. Describe sugar policies impacting food production d. Describe strategies to identify nutrition misinformation
	*Conceptual*: Relationships among food availability, food access, food marketing, social determinants of health, and oral disease; Associations between nutrition misinformation and oral disease	*Understand*: Interpreting and inferring *Analyze*: Differentiate	a. Describe systemic barriers to healthy food choices b. Describe how nutrition misinformation increases the risk of oral disease
	*Procedural*:		
	*Metacognitive*: Appreciation of how food policies and/or nutrition misinformation impacts oral disease risk	*Create*: Assembling and planning	a. Propose strategies to address food policies associated with increased oral disease b. Design educational strategies to address nutrition misinformation
Diet assessment and counseling	*Factual*: Dietary screening protocol for oral disease risk; Dietary assessment protocol for oral disease risk within the scope of practice; Diet counseling strategies; Referral resources	*Remember*: Recognize and retrieve *Understand*: Interpreting, summarizing, and inferring	a. Identify primary dietary risk factors for oral disease b. Describe strategies for negotiating better dietary options with patients c. Identify referrals for in‐depth dietary counseling, food access, and/or other food‐related barriers to healthy eating
	Conceptual: Relationships among dietary risk factors, food values and preferences, environmental barriers to healthier diets, and capacity for change; Integration of listening skillset, prioritization of diet‐related concerns, and negotiation of dietary care plan	*Understand*: Inferring, comparing, and explaining *Analyze*: Differentiating and organizing	a. Recognize dietary risk factors within the context of the patient's food values, food preferences, and environmental barriers b. Identify realistic dietary changes and strategies to achieve changes
	*Procedural*: Screening and/or assessment of the patient's diet, eating behaviors, food security status, and environmental barriers within the scope of practice; Counseling on food choices and eating behaviors within the scope of practice	*Apply*: Executing	a. Screen healthy patients for food choices and/or eating behaviors that increase risk of oral disease b. Assess diets of patients with oral disease to identify food choices and/or eating behaviors contributing to oral disease c. Negotiate better food choices and eating behaviors with patients and provide strategies for behavior change
	*Metacognitive*: Integration of patient food choices, food values, food preferences, eating behaviors, oral function, and social determinants of health within the environmental context with oral and systemic disease risk	*Create*: Assembling and planning	a. Identify relevance of patient food choices and eating behaviors for oral and/or systemic disease b. Identify resources and/or strategies to address barriers to healthy eating c. Outline a dietary plan to reduce oral and systemic disease

Following identification of the educational objectives, we identified behavior expectations and evaluation criteria to establish diet‐related oral health competencies (Table 2). For example, a competent student would be expected “to identify and address diet‐related caries risk factors within the context of environmental and social barriers.” Evaluation criteria for competency include the ability to screen and assess diet‐related caries risk factors, screen for barriers to healthy food intake, negotiate dietary recommendations to reduce caries risk, and refer patients as appropriate.

**TABLE 2:** Behavior expectations for oral health care practitioners and evaluation criteria to establish diet‐related oral health competency.

Oral health domain	Behavior expectation	Outcome evaluation criteria
Dental caries	1. Identifies and addresses diet‐related caries risk factors (e.g., foods and eating behaviors) within the context of environmental and social barriers	1. Screens healthy patients for diet‐related caries risk factors *and* 2. Assesses the diet of patients with active caries and/or diet‐related caries risk factors *and* 3. Screens for oral dysfunction, food insecurity, and/or environmental barriers to healthy food intake *and* 4. Negotiates dietary recommendations to reduce caries risk and support systemic health with patient *and* 5. Refers patients with significant dietary needs to a registered dietitian *and* 6. Refers patients with food‐related environmental barriers to the appropriate community partner
Dental erosion	1. Identifies and addresses dietary sources of acid and eating behaviors that increase the risk of dental erosion 2. Identifies behaviors associated with and/or risk factors consistent with an eating disorder	1. Screens patients with erosion for eating disorders, gastroesophageal reflux, and rumination syndrome *and* 2. Refers patients with suspected eating disorders, gastroesophageal reflux, or rumination syndrome to their primary health care provider or appropriate medical specialist *and* 3. Assesses diet of patients with erosion for eating behaviors, prolonging exposure to acidic foods and beverages
Periodontal disease	1. Identifies and addresses diets of low nutrient density and/or high energy density, increasing risk of periodontal disease in the context of environmental and social barriers	1. Screens patients for food group intakes relative to dietary guideline recommendations *and* 2. Identifies patients with inappropriate weight gain *and* 3. Screens for oral dysfunction, food insecurity, and/or environmental barriers to intake of healthy foods *and* 4. Negotiates dietary recommendations with patients to improve food group intakes and/or reduce energy intake consistent with systemic health *and* 5. Refers patients with significant dietary needs to a registered dietitian *and* 6. Refers patients with food‐related environmental barriers to an appropriate community partner
Oral cancer	1. Identifies and addresses diet‐related risk factors for oral cancer in the context of environmental and social barriers	1. Screens patients for food group intakes relative to dietary guideline recommendations *and* 2. Screens patients for alcohol and fried food intakes *and* 3. Screens for oral dysfunction, food insecurity, and/or environmental barriers to intake of healthy foods *and* 4. Negotiates dietary recommendations with patients to improve food group intakes and lower alcohol and fried food intakes *and* 5. Refers patients with significant dietary needs to a registered dietitian *and* 6. Refers patients with food‐related environmental barriers to an appropriate community partner
Oral dysfunction	1. Identifies oral factors (i.e., tooth loss, denture use, soft tissue loss, pain, xerostomia, and/or dysphagia) that interfere with the expected eating process 2. Recommends food and/or beverage modifications addressing oral factors to ensure appropriate food intakes	1. Screens for oral dysfunction *and* 2. Identifies the impact of oral dysfunction on food and beverage intake *and* 3. Provides strategies to modify textures and/or meal patterns to ensure adequate food/beverage intakes *and* 4. Refers patients with suspected dysphagia to their primary health care provider or appropriate medical specialist *and* 5. Refers patients with significant dietary needs to registered dietitian

## 4 Discussion

Nutrition is a complex science fundamental to human health. While a lack of nutrients limits growth and tissue health, an excess or imbalance of nutrients contributes to metabolic dysfunction and increases the risk of chronic diseases. Oral health—that is, the development and maintenance of orofacial structures supporting oral function—depends on appropriate nutrition. Knowledge of dietary behaviors and nutrition is essential for OHCPs to promote oral health and prevent oral disease. Training OHCSs on diet and nutrition principles during their undergraduate training is necessary to enable OHCPs to provide dietary counseling within their scope of practice. Historically, diet‐ and nutrition‐related oral health education has been limited by the absence of competency criteria to inform a curriculum. We have proposed a framework for diet‐related oral health competencies for graduating OHCSs. Ideally, the framework will act as a foundation to further refine competencies appropriate to guide diet‐related oral health curricula to promote oral, systemic, and nutritional health.

We began our process by identifying diet and nutrition‐related oral health domains—the point where oral health intersects with dietary behaviors. We then identified the diet and nutrition principles supporting these domains. Finally, we identified the behavioral concepts necessary to support dietary counseling for oral health. We used Bloom's knowledge dimensions to identify the type or level of knowledge needed by OHCSs as well as the cognitive processes required to support that knowledge dimension [10]. Educational objectives were outlined based on the knowledge and cognitive process dimensions for each diet‐related oral health domain, diet and nutrition principle, and dietary counseling component. Diet‐ and nutrition‐related oral health content can be integrated into existing oral health curricula via online instruction, integration into existing coursework, or through nutrition courses with clinic integration [6, 8, 37]. Course objectives and learning outcomes consistent with educational objectives are defined during curricular development. Interprofessional instruction engaging both nutrition and oral health care faculty is encouraged to bridge disciplines and maximize student education and training.

Competency documentation requires a successful demonstration of the expected behavior. For each oral health domain, we assimilated the educational objectives to articulate the behavioral expectations consistent with competency and identified the associated evaluation criteria. Competency documentation is best performed in clinical settings during patient care and may be supervised by nutrition (registered dietitians) or oral health care faculty. Engagement of nutrition faculty and social workers in clinic models interprofessional patient care and encourages future collaboration.

Throughout the process, we recognized that OHCPs have multiple responsibilities and are not nutrition professionals. The breadth and depth of nutrition science is overwhelming, and we sought to identify “need to know” diet and nutrition knowledge and dietary behaviors to support oral health. In doing so, we recognized the OHCP's scope of practice, identified resource rationales and sources, and encouraged interprofessional collaboration.

## 5 Conclusion

To achieve the WHO's vision of oral health, the OHCPs must appreciate diet‐related oral disease and address food‐related behaviors to support oral, systemic, and nutritional health. Preparing the OHCS for the future requires a commonly agreed‐upon diet and nutrition knowledge base and behavioral expectations to establish competency. Herein, we present a framework to identify this knowledge base and define diet‐ and nutrition‐related oral health competencies.

## Author Contributions

T.A.M. conceptualized and designed the study, assisted with data collection and interpretation, drafted the initial manuscript, and reviewed and revised the manuscript. R.T.D. and M. C. conceptualized the study, assisted with data interpretation, and critically reviewed the manuscript.

## Funding

The authors have nothing to report.

## Ethics Statement

The IRB Chair at the University of Iowa determined that this project did not meet the regulatory definition of human subjects’ research and did not require review by the IRB (202510185).

## Conflicts of Interest

The authors declare no conflicts of interest.

## References

[1] World Health Organization , “Oral Health,” accessed October 17, 2025, https://www.who.int/health‐topics/oral‐health/#tab=tab_1.

[2] J. Fisher , R. Berman , K. Buse , et al., “Achieving Oral Health for All Through Public Health Approaches, Interprofessional, and Transdisciplinary Education,” NAM Perspectives 2023 (2023): 10–31478, 10.31478/202302b. PMC10238101 37273458

[3] T. A. Marshall and R. Touger‐Decker , “Oral Health and Multimorbidity: Is Diet the Chicken or the Egg?,” Proceedings of the Nutrition Society (2024): 1–8, 10.1017/S0029665124004683. 38742385

[4] Y. L. Kapila , “Oral Health's Inextricable Connection to Systemic Health: Special Populations Bring to Bear Multimodal Relationships and Factors Connecting Periodontal Disease to Systemic Diseases and Conditions,” Periodontology 2000 87 (2021): 11–16, 10.1111/prd.12398. 34463994 PMC8457130

[5] T. Marshall and R. Touger‐Decker . “Oral Health and Disease,” in Modern Nutrition in Health and Disease, 12th ed., ed. K. Tucker (Jones & Bartlett Learning LLC, 2025).

[6] C. E. Fernández , M. J. Torre , C. J. Vargas , C. A. Aravena , J. Santander , and T. A. Marshall , “Diet and Nutrition Integration in Dental Education: A Scoping Review,” European Journal of Dental Education (Early View): 1–14, 10.1111/eje.13136. 40474496

[7] M. Kataoka , L. A. Adam , L. E. Ball , J. Crowley , and R. M. Mclean , “Nutrition Education and Practice in University Dental and Oral Health Programmes and Curricula: A Scoping Review,” European Journal of Dental Education 29 (2025): 64–83, 10.1111/eje.13045. 39473077 PMC11730457

[8] R. Touger‐Decker , “Nutrition Education of Medical and Dental Students: Innovation Through Curriculum Integration,” American Journal of Clinical Nutrition 79 (2004): 198–203, 10.1093/ajcn/79.2.198. 14749223

[9] R. Touger‐Decker and C. Mobley , “Position of the Academy of Nutrition and Dietetics: Oral Health and Nutrition,” Journal of the Academy of Nutrition and Dietetics 113 (2013): 693–701, 10.1016/j.jand.2013.03.001. 23601893

[10] L. W. Anderson and D. R. Krathwohl , A Taxonomy for Learning, Teaching, and Assessing: A Revision of Bloom's, 1st ed. (New York: Longman 2001).

[11] S. E. Dreyfus , “The Five‐Stage Model of Adult Skill Acquisition,” Bulletin of Science, Technology & Society 24 (2004): 177–181, 10.1177/0270467604264992.

[12] P. J. Moynihan and S. A. M. Kelly , “Effect on Caries of Restricting Sugars Intake,” Journal of Dental Research 93 (2014): 8–18, 10.1177/0022034513508954. 24323509 PMC3872848

[13] C. Delaney , J. Warren , O. A. Rysavy , and T. Marshall , “Dietary Questions in Caries Risk Assessment and Their Relationship to Caries,” Journal of Public Health Dentistry 85 (2024): 197–202, 10.1111/jphd.12647. 39377152 PMC12147404

[14] T. S. Carvalho and A. Lussi , “Acidic Beverages and Foods Associated With Dental Erosion and Erosive Tooth Wear,” in Monographs in Oral Science 28 (2020): 91–98. 31940633 10.1159/000455376

[15] E. A. O'Sullivan and M. E. Curzon , “A Comparison of Acidic Dietary Factors in Children With and Without Dental Erosion,” Journal of Dentistry for Children 67 (2000): 186–192. 10902077

[16] X. Shi , P. Zhu , M. Du , et al., “Dietary Patterns and Periodontitis: A Systematic Review,” Journal of Periodontal Research 60 (2025): 300–314, 10.1111/jre.13346. 39248151

[17] L. M. Jauhiainen , P. V. Ylöstalo , M. Knuuttila , S. Männistö , N. Kanerva , and A. L. Suominen , “Poor Diet Predicts Periodontal Disease Development in 11‐Year Follow‐Up Study,” Community Dentistry and Oral Epidemiology 48 (2020): 143–151, 10.1111/cdoe.12513. 31868961

[18] N. Mi , M. Zhang , Z. Ying , X. Lin , and Y. Jin , “Vitamin Intake and Periodontal Disease: A Meta‐Analysis of Observational Studies,” BMC Oral Health 24 (2020): 117, 10.1186/s12903-024-03850-5. PMC10799494 38245765

[19] J. Rodríguez‐Molinero , B. C. Migueláñez‐Medrán , C. Puente‐Gutiérrez , et al., “Association Between Oral Cancer and Diet: An Update,” Nutrients (1229), 10.3390/nu13041299. PMC8071138 33920788

[20] S. Cirmi , M. Navarra , J. V. Woodside , and M. M. Cantwell , “Citrus Fruits Intake and Oral Cancer Risk: A Systematic Review and Meta‐Analysis,” Pharmaceutical Research 133 (2018): 187–194. 10.1016/j.phrs.2018.05.008 29753688

[21] R. Shrivastava , A. Gupta , N. Mehta , D. Das , and A. Goyal , “Dietary Patterns and Risk of Oral and Oropharyngeal Cancers: A Systematic Review and Meta‐Analysis,” Cancer Epidemiology 93 (2004): 102650, 10.1016/j.canep.2024.102650. 39226679

[22] S. Honeywell , H. Samavat , R. Touger‐Decker , J. S. Parrott , E. Hoskin , and R. Zelig , “Associations Between Dentition Status and Nutritional Status in Community‐Dwelling Older Adults,” JDR Clinical & Translational Research 8 (2023): 93–101, 10.1177/23800844211063859. 35000489

[23] T. Lahoud , A. Y.‐D. Yu , and S. King , “Masticatory Dysfunction in Older Adults: A Scoping Review,” Journal of Oral Rehabilitation 50 (2023): 724–737, 10.1111/joor.13493. 37183339

[24] R. Zelig , S. Goldstein , R. Touger‐Decker , et al., “Tooth Loss and Nutritional Status in Older Adults: A Systematic Review and Meta‐Analysis,” JDR Clinical & Translational Research 7 (2022): 4–15, 10.1177/2380084420981016. 33345687

[25] U.S. Department of Agriculture and U.S. Department of Health and Human Services , “Dietary Guidelines for Americans,” in DietaryGuidelines.Gov, 9th ed. (U.S. Department of Agriculture and U.S. Department of Health and Human Services, 2020).

[26] “MyPlate,” United States Department of Agriculture , accessed October 17, 2025, https://www.myplate.gov/.

[27] S. P. Murphy , A. A. Yates , S. A. Atkinson , S. I. Barr , and J. Dwyer , “History of Nutrition: The Long Road Leading to the Dietary Reference Intakes for the United States and Canada,” Advances in Nutrition 7 (2016): 157–168, 10.3945/an.115.010322. 27180379 PMC4717892

[28] F. Pamuk and A. Kantarci , “Inflammation as a Link Between Periodontal Disease and Obesity,” Periodontology 2000 90 (2022): 186–196, 10.1111/prd.12457. 35916870

[29] F. Feillet and C. Agostoni , “Nutritional Issues in Treating Phenylketonuria,” Journal of Inherited Metabolic Disease 33 (2010): 659–664, 10.1007/s10545-010-9043-4. 20151202

[30] M. I. Pinto‐Sanchez , J. J. Blom , P. R. Gibson , and D. Armstrong , “Nutrition Assessment and Management in Celiac Disease,” Gastroenterology 167 (2024): 116–131. 38593924 10.1053/j.gastro.2024.02.049

[31] E. J. Bandt , D. Mozaffarian , C. W. Leung , S. A. Berkowitz , and V. L. Murthy , “Diet and Food and Nutrition Insecurity and Cardiometabolic Disease,” Circulation Research 132 (2023): 1692–1706. 37289902 10.1161/CIRCRESAHA.123.322065 PMC10882643

[32] World Health Organisation . “Sustainable Healthy Diets: Guiding Principles.” (Food and Agriculture Organization of the United Nations, 2019).

[33] M. Springmann , K. Wiebe , D. Mason‐D'croz , T. B. Sulser , M. Rayner , and P. Scarborough , “Health and Nutritional Aspects of Sustainable Diet Strategies and Their Association With Environmental Impacts: A Global Modelling Analysis With Country‐Level Detail,” Lancet Planetary Health 2 (2018): e451–e461, 10.1016/S2542-5196(18)30206-7. 30318102 PMC6182055

[34] M. Perignon , G. Masset , G. Ferrari , et al., “How Low Can Dietary Greenhouse Gas Emissions be Reduced Without Impairing Nutritional Adequacy, Affordability and Acceptability of the Diet? A Modelling Study to Guide Sustainable Food Choices,” Public Health Nutrition 19 (2016): 2662–2674, 10.1017/S1368980016000653. 27049598 PMC10448381

[35] T. Durá‐Travé and F. Gallinas‐Victoriano , “Dental Caries in Children and Vitamin D Deficiency: A Narrative Review,” European Journal of Pediatrics 183 (2004): 523–528, 10.1007/s00431-023-05331-3. PMC10912272 37966493

[36] D. Kelly , T. O'dowd , and U. Reulbach , “Use of Folic Acid Supplements and Risk of Cleft Lip and Palate in Infants: A Population‐Based Cohort Study,” British Journal of General Practice 62 (2012): e466–e472, 10.3399/bjgp12X652328. PMC3381272 22781994

[37] M. Crowe , M. O'Sullivan , L. Winning , et al., “Implementation of a Food Science and Nutrition Module in a Dental Undergraduate Curriculum,” European Journal of Dental Education 27 (2023): 402–408. 35582770 10.1111/eje.12822

